# Clinimetrics of the Italian version of the Montreal Cognitive Assessment (MoCA) in adult-onset idiopathic focal dystonia

**DOI:** 10.1007/s00702-023-02663-0

**Published:** 2023-06-12

**Authors:** Alfonsina D’Iorio, Edoardo Nicolò Aiello, Assunta Trinchillo, Vincenzo Silani, Nicola Ticozzi, Andrea Ciammola, Barbara Poletti, Marcello Esposito, Gabriella Santangelo

**Affiliations:** 1 Department of Psychology, University of Campania “Luigi Vanvitelli”, Caserta, Italy; 2 Department of Neurology and Laboratory of Neuroscience, IRCCS Istituto Auxologico Italiano, Milan, Italy; 3 Clinical Neurophysiology Unit, Cardarelli Hospital, Naples, Italy; 4 Department of Neurosciences, Reproductive Sciences and Odontostomatology, University of Naples Federico II, Naples, Italy; 5 Department of Pathophysiology and Transplantation, “Dino Ferrari” Center, Università Degli Studi di Milano, Milan, Italy

**Keywords:** Montreal Cognitive Assessment, Dystonia, Cognitive screening, Neuropsychology, Movement disorders, Hyperkinetic

## Abstract

This study aimed at assessing the clinimetrics of the Montreal Cognitive Assessment (MoCA) in an Italian cohort of patients with adult-onset idiopathic focal dystonia (AOIFD). *N* = 86 AOIFD patients and *N* = 92 healthy controls (HCs) were administered the MoCA. Patients further underwent the Trail-Making Test (TMT) and Babcock Memory Test (BMT), being also screened via the Beck Depression Inventory-II (BDI-II) and the Dimensional Apathy Scale (DAS). Factorial structure and internal consistency were assessed. Construct validity was tested against TMT, BMT, BDI-II and DAS scores, whilst diagnostics against the co-occurrence of a defective performance on at least one TMT measure and on the BMT. Case–control discrimination was examined. The association between MoCA scores and motor-functional measures was explored. The MoCA was underpinned by a mono-component structure and acceptably reliable at an internal level. It converged towards TMT and BMT scores, as well as with the DAS, whilst diverging from the BDI-II. Its adjusted scores accurately detected cognitive impairment (AUC = .86) at a cut-off of < 17.212. The MoCA discriminated patients from HCs (*p* < .001). Finally, it was unrelated to disease duration and severity, as well as to motor phenotypes. The Italian MoCA is a valid, diagnostically sound and feasible cognitive screener in AOIFD patients.

## Introduction

The non-motor phenotype of patients with adult-onset idiopathic focal dystonia (AOIFD) (Albanese et al. 2013) has been recently acknowledged to be possibly featured by cognitive deficits of a dysexecutive-inattentive and amnestic nature (Aita et al. 2022, Bailey et al. 2022) which are thought to be accounted for by an involvement of both frontal-striatal networks (Kuyper et al. 2011) and collicular, thalamic and middle-temporal structures (Rafee et al. 2021). Consistently, and even though the functional entailments of such dysfunctions in this population are to this day mostly unknown (Monaghan et al. 2021), cognitive screening has been preventively recommended in AOIFD patients (Yang et al. 2017), especially to the aim of monitoring their cognitive status within the context of either surgical of pharmacological treatments (Jahanshahi 2011, Jahanshahi 2017).

However, no consensus has been reached to this day as to which cognitive screener(s) might be suitable for use in this population–since available studies on cognition in AOIFD employed such tests solely to semiological aims, whilst neglecting the assessment of their clinimetrics and feasibility (Bailey et al. 2022). However, in this respect, promising evidence has been delivered on the psychometric soundness and cross-sectional feasibility of the Montreal Cognitive Assessment (MoCA) (Nasreddine et al. 2005) in patients with a genetic dystonia-parkinsonim syndrome (Aliling et al. 2019)–which present with neural alterations and thus cognitive features similar to AOIFD patients (Jamora et al. 2014). Relatedly, the vast amount of findings that favor the use of the MoCA in other extrapyramidal disorders (Julayanont et al. 2017) further bolsters the rationale underlying the exploration of the clinimetrics and feasibility of such a screener in AOIFD.

Hence, the present study aimed at assessing, in an Italian cohort of AOIFD patients, 1) the psychometrics of the MoCA, 2) its diagnostics within a case-finding setting and 3) its capability to discriminate AOIFD patients from healthy controls (HCs).

## Methods

### Participants

Data on *N* = 86 clinically diagnosed AOIFD patients (Albanese et al. 2013) consecutively referred to Cardarelli Hospital (Naples, Italy) between 2017 and 2023 were retrospectively collected. Additionally, *N* = 92 HCs were prospectively recruited at IRCCS Istituto Auxologico Italiano. Patients and HCs were free of 1) (further) neurological/psychiatric disorders, 2) severe and/or unstable metabolic/internal diseases or system/organ failure and 3) uncorrected hearing/vision deficits. Moreover, HCs were not actively taking any psychotropic medications when recruited.

### Materials

Both groups were administered the Italian version of the MoCA (Aiello et al. 2022a).

Additionally, patients were administered a set of second-level cognitive tests tapping on executive-attentive–*i.e.*, the Trail-Making Test-A/-B/-BA (TMT-A/-B/-BA) (Giovagnoli et al. 1996)–and mnestic functions–*i.e.*, the Babcock Memory Test (BMT) (Novelli et al. 1986), also undergoing a behavioural evaluation via the Beck Depression Inventory-II (BDI-II; *range* = 0–63) (Sica et al. 2007) and the Italian Dimensional Apathy Scale (I-DAS; *range* = 0–72) (Santangelo et al. 2017). The present BMT version, by Novelli et al*.* (Novelli et al. 1986), addresses as the outcome the mean between the number of items immediately recalled and that of items non-incidentally recalled after a 10’-delay (*range* = 0–28).

In patients with blepharospasm, cervical dystonia and laryngeal dystonia, disease severity was assessed via the Jankovic’s Rating Scale (JRS; *range* = 0–8) (Jankovic and Orman 1987), Tsui’s Scale (TS; *range* = 0–25) (Tsui et al. 1986) and Voice Handicap Index (VHI; *range* = 0–120) (Jacobson et al. 1997), respectively. Additionally, patients with hand dystonia were classified as having either task-specific or task-nonspecific disturbances, whilst those with oromandibular dystonia were classified as either jaw-closing or jaw-opening.

### Statistics

Normality checks were run on raw variables by assessing skewness and kurtosis values (judged as abnormal if >|1| and |3|, respectively (Kim 2013)), as well as by visually inspecting histograms and Q-Q plots. Based on this assumption being met or not, either parametric or non-parametric techniques were adopted to test associations/predictions of interest.

In patients, the factorial structure and internal consistency of the MoCA were explored via a Principal Component Analysis and McDonald’s ω coefficient, respectively, whilst its construct validity was tested against the abovementioned cognitive and behavioural measures via Bonferroni-corrected Spearman’s coefficients. An additional set of Bonferroni-corrected Spearman’s correlations was then performed to test the association between the second-level tests and each MoCA subscale–with item being grouped according to Santangelo et al*.* (2015).

Receiver-operating characteristics (ROC) analyses was run to test the diagnostics of both raw and age- and education-adjusted MoCA scores (Aiello et al. 2022a). For this purpose, the positive state–*i.e.*, the occurrence of dysexecutive-inattentive and amnestic-like cognitive dysfunctions–was operationalized as the co-occurrence of a below-cutoff on the BMT (Novelli et al. 1986) and on at least one TMT measure (Giovagnoli et al. 1996). Within such analyses, sensitivity (Se), specificity (Sp), positive and negative predictive values (PPV; NPV) and likelihood ratios (LR + ; LR−) were computed at the optimal cut-off identified via Youden’s *J* statistic. Additionally, the number needed for screening utility (NNSU) was computed as 1/[(Se*PPV) + (Sp*NPV)]–with values 1.02 ≤ meaning that less than ≈1 individual needs to be screened for the test to be useful in the view of ruling-in/ruling-out the occurrence of a positive state (Larner 2019).

Case–control discrimination was tested via a logistic regression by addressing raw MoCA scores as the predictor and group as the outcome; since the two groups were matched for sex (χ^2^(1) = 0.46; *p* = 0.495) but not for age (*t*(176) = -3.97; *p* < 0.001) and education (*t*(166.35) = 4.34; *p* < 0.001), these last two demographics were entered as covariates.

Finally, a set of explorative analyses were run in order to test whether MoCA scores could be confounded by patients’ motor features. First, a Spearman’s coefficient was computed for the association between disease severity (in years) and adjusted MoCA scores. Second, for patients with blepharospasm, cervical dystonia and laryngeal dystonia, Bonferroni-corrected Pearson’s correlations were run between disease severity measures (*i.e.*, JRS, TS and VHI score) and adjusted MoCA scores. Third, an *F*-tests was run to compare adjusted MoCA scores scores across motor phenotypes (*i.e.*, blepharospasm *vs.* cervical dystonia *vs.* hand dystonia *vs.* laryngeal dystonia *vs.* oromandibular dystonia *vs.* lower limb dystonia).

Analyses were performed via jamovi 2.3, R 4.1 and IBM SPSS 27. Missing data were excluded pairwise.

## Results

Participants’ background, clinical and neuropsychological measures are summarized in Table 1.

**Table 1:** Participants’ background, clinical and cognitive measures

	AOIFD	HCs	*p*
*N*	86	92	–
Sex (male/female)	25/63	30/62	0.495^a^
Age (years)	60.1 ± 11 (25–83)	53.5 ± 11 (24—86)	<0 .001^b^
Education (years)	10.2 ± 4.5 (3–18)	13 ± 3.8 (5–18)	0.001^b^
Disease duration (years)	8.7 ± 7.3 (1–85)	–	–
Phenotype (%)			
Blespharospasm	42%	–	–
Cervical dystonia	36%	–	–
Hand dystonia	8%	–	–
Laryngeal dystonia	7%	–	–
Oromandibular dystonia	5%	–	–
Lower limb dystonia	2%	–	–
MoCA	21.3 ± 4.1 (11–29)	27.1 ± 2.5 (18–30)	<0 .001^b^
Below-cut-off (%)^c^	22%	–	–
TMT			
Part A	52.6 ± 30.8 (13–185)	–	–
Part B	173.2 ± 107.9 (28—490)	–	–
Part B-A	121.7 ± 86.6 (18–388)	–	–
BMT	10.6 ± 4.6 (1–20.5)	–	–
BDI-II	8.7 ± 9.9 (0–44)	–	–
DAS	23.2 ± 10.4 (3–54)	–	–

*AOIFD* adult-onset idiopathic focal dystonia, *HCs* healthy controls, *MoCA* Montreal Cognitive Assessment, *TMT* Trail-Making Test, *BMT* Babcock Memory Test, *BDI-II* Beck Depression Inventory-II, *DAS* Dimensional Apathy Scale. ^a^χ^2^-statistic; ^b^*t*-statistic; ^c^Aiello et al*.* (2022a)

The MoCA proved to be underpinned by a mono-component structure accounting for 38.41% of variance; all MoCA subscales substantially loaded on such a component (*range* = 0.58–0.79), except for the MoCA-Orientation (0.24). Internal consistency was acceptable (McDonald’s ω = 0.67). At α_adjusted_ = 0.008, MoCA scores converged with both the BMT (*r*_*s*_(86) = 0.66; *p* < 0.001) and all TMT measures (− 0.67 ≤ *r*_*s*_(86) ≤ − 0.55; *p*s < 0.001), as well as with the DAS (*r*_*s*_(82) = − 0.37; *p* < 0.001)–whilst diverging from the BDI-II (*r*_*s*_(83) = − 0.11; *p* = 0.326). *Executive Functioning*, *Visuo-spatial*, *Attention* and *Memory* MoCA subscales proved to be significantly associated with both TMT measures and the BMT, whilst this was not true for those the MoCA-Orientation subscale (Table 2); the MoCA-Language proved to be related to the BMT only (Table 2).

**Table 2:** Spearman’s coefficients between the second-level tests and MoCA subscales

	MoCA-EF	MoCA-VS	MoCA-L	MoCA-A	MoCA-M	MoCA-O
TMT-A						
* r*_*s*_	− 0.47	− 0.36	− 0.21	− 0.47	− 0.37	− 0.11
* p*	< .001	0.002	0.057	< 0.001	< 0.001	0.304
TMT-B						
* r*_*s*_	− 0.53	− 0.56	− 0.26	− 0.56	− 0.39	− 0.12
* p*	< 0.001	< 0.001	0.014	< 0.001	< 0.001	0.281
TMT-BA						
* r*_*s*_	− 0.48	− 0.56	− 0.21	− 0.51	− 0.34	− 0.09
* p*	< 0.001	< 0.001	0.048	< 0.001	0.001	0.423
BMT						
* r*_*s*_	0.49	0.43	0.38	0.44	0.50	0.05
*p*	< 0.001	< 0.001	< 0.001	< 0.001	< 0.001	0.655

*MoCA* Montreal Cognitive Assessment, *EF* Executive functioning, *VS* Visuospatial, L Language, *A* Attention, *M* Memory, *O* Orientation, *TMT* Trail-Making Test, *BMT* Babcock Memory Test. Coeffcients in bold are significant at α_adjusted_ = 0.003. MoCA items were grouped according to Santangelo et al. (2015)

**Fig. 1:**
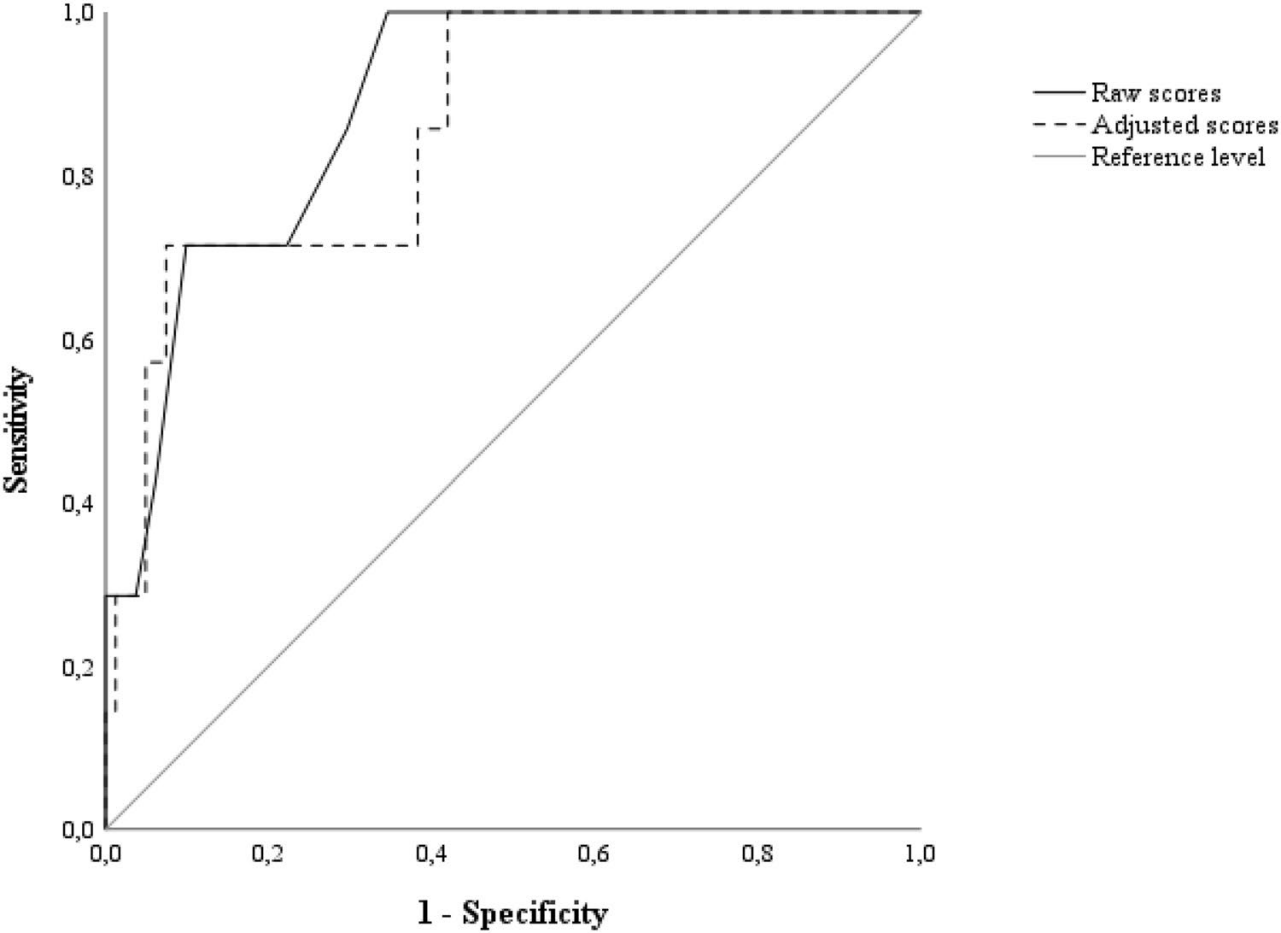
ROC curves for raw and adjusted MoCA scores. *ROC* receiver-operating characteristics, *MoCA* Montreal Cognitive Assessment. Raw scores (cut-off: ≤ 20): *AUC *= 0.89; *SE* = 0.05; CI 95% [0.79, 0.99]; adjusted scores (cut-off: < 17.212): AUC = 0.86; *SE* = 0.07; CI 95% [0.74, 0.99]. MoCA scores were adjusted according to Aiello et al*.* (2022a)

Seven patients (8%) were classified as cognitively impaired according to the present operationalization. In identifying such patients, both raw and adjusted MoCA scores proved to be highly accurate (Fig. 1), as well as to be featured, at the optimal cutoffs of ≤ 20 (*J* = 0.66) and < 17.212 (*J* = 0.63), respectively. However, diagnostic metrics proved to be slightly better and by far more balanced for adjusted scores (Se = 0.71; Sp = 0.92; PPV = 0.46; NPV = 0.97; LR + = 9.41; LR- = 0.31; NNSU = 0.82) when compared to raw ones (Se = 1; Sp = 0.66; PPV = 0.21; NPV = 1; LR + = 2.93; LR- = 0; NNSU = 1.15). According to the raw and adjusted cutoffs, 40% and 13% of patients were classified as impaired on the MoCA, respectively. Interestingly, the classification yielded by the present adjusted cutoff agreed to a greater extent with that resulting from the application of the normality threshold derived within the latest Italian normative study–*i.e.*, < 18.59 (Aiello et al. 2022a)–(Cohen’s *k* = 0.68; agreement rate: 91%) than with that yielding from the disease-specific, raw cutoff herewith derived (Cohen’s *k* = 0.37; agreement rate: 73%).

Net of age and education, MoCA scores proved to be able to discriminate patients from HCs (*z* = − 5.99; OR = 0.50, CI 95% [0.40, 0.62]; *p* < 0.001) with a classification accuracy of 80% (AUC = 0.90; Se = 0.86; Sp = 0.74).

Table 3 displays patients’ demographic and clinical features, as well as MoCA scores, across different motor phenotypes. The MoCA proved to be unrelated to disease duration (*r*_*s*_(85) = 0.01; *p* = 0.917). At α_adjusted_ = 0.017, no association was detected between the MoCA and the JRS in blepharospasm patients (*r*_*s*_(36) = − 0.24; *p* = 0.157), the TS in cervidal dystonia patients (*r*_*s*_(31) = − 0.30; *p* = 0.106) or the VHI in laryngeal dystonia patients (*r*_*s*_(6) = − 0.50; *p* = 0.317). Finally, MoCA scores did not vary based on motor phenotypes (*F*(5, 80) = 0.21; *p* = 0.956).

**Table 3:** Patients’ demographic, clinical and cognitive measures across motor phenotypes

	BSP	CD	HD	LD	OMD	LLD	*p*
*N*	36	31	7	6	4	2	–
Sex (male/female)	3/33	16/15	3/4	1/5	1/3	0/2	0.004^a^
Age (years)	64.3 ± 8.3 (47–83)	56.1 ± 13 (25–72)	55 ± 9.1 (41–67)	62.6 ± 10.5 (46–72)	57.7 ± 11.2 (43–67)	59 ± 2.8 (57–61)	0.136^b^
Education (years)	9.4 ± 4.6 (3–18)	11.1 ± 4.4 (5–18)	12 ± 4.5 (8–18)	9.1 ± 4.4 (5–16)	9.5 ± 3.6 (5–13)	9 ± 5.6 (5–13)	0.722^b^
Disease duration (years)	6.2 ± 3.9 (1–14)	11.3 ± 11.6 (1–45)	10.1 ± 7.3 (3–21)	6.3 ± 6.2 (1–17)	9.3 ± 9.2 (1–21)	11.5 ± 6.4 (7–16)	0.400^c^
Jankovic’s Rating Scale	5.7 ± 1.1 (2–8)	–	–	–	–	–	–
Tsui’s Scale	–	9.7 ± 2.9 (4–15)	–	–	–	–	–
Voice Handicap Index	–	–	–	82.5 ± 5.5 (75–90)	-	-	-
Task-specificity (*N*)	–	–	5	–	–	–	–
Jaw-opening/-closing (*N*)	–	–	–	–	1/3	–	–
MoCA							
Total	20.6 ± 3.8 (12–27)	22.1 ± 4.4 (11–29)	21.5 ± 3.9 (16–26)	20.1 ± 2.9 (17–25)	22 ± 3.3 (18–26)	20 ± 8.4 (14–26)	0.799^c^
Below-cut-off (*N*)^d^	5	3	2	0	0	1	0.324^a^
Executive Functioning	2 ± 1.1 (0–4)	2.1 ± 1.4 (0–4)	1.5 ± 0.9 (0–3)	2.1 ± 0.7 (1–3)	2.2 ± 0.9 (1–3)	2 ± 2.8 (0–4)	0.867^b^
Visuospatial	2.5 ± 0.9 (1–4)	2.9 ± 1.1 (0–4)	2.5 ± 0.9 (1–4)	1.6 ± 0.8 (1–3)	2.7 ± 0.9 (2–4)	2 ± 0 (2–2)	0.154^b^
Language	4.3 ± 1.2 (1–6)	4.5 ± 1.1 (1–6)	4.5 ± 1.1 (3–6)	4.3 ± 1.2 (3–6)	4.2 ± 0.9 (3–5)	5 ± 1.4 (4–6)	0.974^b^
Attention	4.6 ± 1.2 (1–6)	5 ± 1.4 (1–6)	4.7 ± 1.4 (2–6)	4.6 ± 0.5 (4–5)	5.7 ± 0.5 (5–6)	3 ± 2.8 (1–5)	0.105^c^
Memory	1.7 ± 1.5 (0–5)	2 ± 1.4 (0–5)	2.5 ± 0.7 (1–3)	1.5 ± 1.3 (0–3)	1 ± 1.4 (0–3)	3.5 ± 0.7 (3–4)	0.136^b^
Orientation	5.8 ± 0.4 (4–6)	5.8 ± 0.5 (4–6)	6 ± 0 (6–6)	6 ± 0 (6–6)	6 ± 0 (6–6)	5 ± 1.4 (4–6)	0.272^c^

*BSP* blepharospasm, *CD* cervical dystonia, *HD* hand dystonia, *LD* laryngeal dystonia, *OMD* oromandibular dystonia, *LLD* lower limb dystonia, *MoCA* Montreal Cognitive Assessment. ^a^χ^2^-statistic; ^b^*F*-statistic; ^c^χ^2^-statistic (Kruskal–Wallis test); ^d^adjusted MoCA score (Aiello et al. 2022a) < 17.212

## Discussion

The present study provides, for the first time within the international literature, a detailed report on the clinimetric soundness and feasibility of the MoCA in AOIFD patients–by also delivering Italian practitioners and clinical researchers with disease-specific cutoffs for such a screener in this population.

As to its psychometrics, the MoCA herewith proved to (1) be overall featured by a mono-component structure and (2) be acceptably reliable at an internal level–this supporting the notion of it capturing global cognitive efficiency, as well as (3) to converge with second-level measures of executive-attentive (*i.e.*, the TMT) and mnestic functions (*i.e.*, the BMT). This last finding is in agreement with the notion of the MoCA heavily loading on such cognitive domains/functions (Aiello et al. 2022a), being also supported by the fact that, within this study, MoCA subscales tapping on attention, executive functions and executive-based visuospatial skills, as well as memory, were significantly associated with both the TMT and the BMT. Taken together, such findings support the use of the MoCA in AOIFD patients–whose cognitive phenotype is indeed not infrequently characterized by dysexecutive-inattentive and amnestic features (Aita et al. 2022).

Moreover, within the present study, the MoCA was found to diverge from depression levels, by nevertheless being associated with apathetic features. Whilst such findings are, to the best of the Authors’ knowledge, unprecedented, that on cognitive performances being inversely related to apathetic, but not depressive, features is in line with the literature on other extrapyramidal disorders of a degenerative etiology–*i.e.*, Parkinson’s (Santangelo et al. 2018) and Huntington’s disease (Baudic et al. 2006).

Remarkably, this report demonstrates that the MoCA is able to accurately detect cognitive impairment in AOIFD patients. At the same time, when looking at the diagnostic metrics associated with the raw and adjusted cutoffs herewith derived, the threshold identified on adjusted scores clearly outperformed the raw one at a unitary level–as indexed by the NNSU. Hence, a cut-off of < 17.212 is suggested for the detection of cognitive dysfunction in AOIFD patients.

Moreover, the present investigation confirms that the MoCA is able to accurately discriminate AOIFD patients from HCs. Such a finding, along with the abovementioned ones on the diagnostics of the MoCA within a case-finding scenario, is in contrast with the proposed notion according to which first-level tests would not be sufficient to detect cognitive impairment in AOIFD patients (Aita et al. 2022). However, with this regard, it has to be borne in mind that the MoCA is a screener, thus not conveying any actual diagnostic information per se: indeed, whilst being suitable for a first-level evaluation of patients’ cognitive status, a screening session should be always followed, with a positive test result, by the administration of a second-level cognitive battery.

Finally, this report suggests that the MoCA is not confounded by either disease duration or disease severity, as well as that its scores do not vary as a function of patients’ motor phenotypes.

The above being said, the present study is of course not free of limitations.

First, the MoCA-Orientation proved not to substantially load on the underlying, mono-component factor: however, such a finding is unsurprising, giving that items assessing orientations are commonly susceptible to ceiling effects, and thus featured by a relatively low variability, in non-demented patients. Second, albeit acceptable, the internal consistency of the MoCA was not excellent. Nevertheless, in this respect, it should be borne in mind that indices of internal consistency should not be the preferred choice when assessing the reliability of cognitive screeners–since they often include items tapping on different cognitive domains/functions (Aiello et al. 2022b). Hence, future investigations are encouraged that test the reliability of the MoCA in this population by focusing on other computation models–*e.g.*, at inter-rater and test–retest levels. Indeed, such psychometrics have been not herewith tested due to the retrospective nature of this work–this representing its third limitation. Fourth, it is worth mentioning that, within this study, the PPV associated with the adjusted cutoff proved to be low: however, this is likely accounted for by the fact that such a metric is heavily dependent on the proportion of positive individuals within the study sample–which herewith proved to be, in fact, low (Bossuyt 2010). At variance, likelihood ratios should be given greater attention by users as being information-based diagnostic metrics that are prevalence-independent (Bossuyt 2010).

Fifth, it has to be noted that, at variance with blapherospasms and cervical dystonias, the other phenotypes were herewith under-represented; moreover, data on disease severity were available only for patients with blapherospasm, cervical dystonia and laryngeal dystonia. Hence, such findings have to be regarded as preliminary, and it is advisable that future studies delve with a greater extent of detail into the topic of whether the MoCA is confounded or not by motor-functional features in this population. Relatedly, due to the retrospective nature of this investigation, no real-time information has been delivered on the extent to which motor disabilities could have affected MoCA performances. For instance, patients with upper-limb AOIFD might have been challenged when executing those actions required by constructional praxis tasks, whilst those with blepharospasms might have had difficulties in those tasks relying on visual stimuli (*e.g.*, confrontation-naming items and those included within the MoCA-Visuo-spatial). After all, albeit no motor-functional feature was herewith found to affect MoCA scores, it has to be noted that this test has not been originally intended to be motor-free. Thus, examiners should always ascertain, prior to its administration, that patients’ motor disabilities do not prevent them to undertake the MoCA.

Finally, albeit including measures of depression and apathy, this report was lacking of data on patients’ anxiety levels–which could have confounded their cognitive performances. Hence, further investigations are needed in order to determine whether such a psychopathological feature affects MoCA scores in this population.

In conclusion, the MoCA is a valid, diagnostically sound and feasible cognitive screener in AOIFD patients, whose adoption is thus encouraged in clinical practice and research. An adjusted score (Aiello et al. 2022a) lower than 17.212 should be regarded as suggestive of cognitive impairment by Italian clinicians and researchers.

## Data Availability

Datasets associated with the present study are available upon reasonable request of interested researchers.
